# The Mosquito

**Published:** 1902-04

**Authors:** Ennion G. Williams

**Affiliations:** Richmond, Va., Professor Histology, Pathology and Bacteriology in Medical College of Virginia; Pathologist to Old Dominion Hospital, Etc.


### THE MOSQUITO.*

By ENNION G. WILLIAMS, M.D., Richmond, Va.,

Professor Histology, Pathology and Bacteriology in Medical College of Virginia; Pathologist to Old Dominion Hospital, Etc.

I am not an authority on the mosquito, and the information I am about to impart is derived chiefly from the government reports and that valuable book, "The Mosquito," by Dr. L. O. Howard, our government entomologist. This should be widely distributed in every community where there are mosquitoes and malaria.

Like so many advancements in medicine, the mosquito theory of the transmission of disease has been subjected to the purging fire of ridicule and skepticism. The theory is still in its infancy, and may be modified in the process of further investigation; but the fact is established that the mosquitoes are concerned in the transmission of three diseases, or, in other words, that they are the intermediary hosts in the development of the micro-organisms of malaria, yellow fever and filariasis. Even if this were not true, knowledge of the mosquito might still be within the sphere of the practitioner who is concerned in the promotion of health and comfort as well as in healing the sick. Who can deny that the common culex, with its proclivities for sucking human blood, is not a disturber of the peace and a public enemy, as well as her sister—

*Read before the Richmond Academy of Medicine and Surgery, September 24, 1901.

the murderous villain—the anopheles? A member of the Academy is absent to-night because an abraded mosquito bite furnished an open door to the entrance of pyogenic bacteria.

It is interesting to know that the relation between mosquitoes and malaria was first suggested about twenty years ago by an American physician, Dr. A. F. A. King, and first demonstrated by an Englishman, Dr. Ross; and later on, by the Italians, Bignami, Grassi and Basstianelli, and the German, Koch.

There are about two hundred and fifty known species of mosquities, of which only about thirty have as yet been found in the United States. They are very widely distributed. The gold-seekers of Alaska have as great stories to tell of their numbers and ferocity as the pleasure-seekers of the Jersey coast. A gentleman recently wrote from Alaska that they "existed in countless numbers, driving us to the verge of suicide and insanity." It is said that "in Lapland, their numbers are so prodigous as to be compared to a flight of snow when the flakes fall thickest; or to the dust of the earth. The natives cannot take a mouthful of food, or lie down to sleep in their cabins unless they are fumigated almost to suffocation. In the air, you cannot draw your breath without having your mouth and nostrils filled with them." Indeed, some of the mosquito stories vie in point of credibility with the proverbial fish stories. Perhaps the fact that three of the four stages of their life are spent in water, would justify one in speaking of mosquito stories as fishy. The most classical and historical account of the effects of these insects is that given by Theodoretus, who said that when Sapor, king of Persia, was laying siege to the town of Nisibis, his army—men, elephants and beasts of burden—were attacked by a plague of gnats, compelling him to raise the siege and putting his army to rout.

The life history of the mosquito is divided into four stages—the egg, the larva and the pupa, and the adult mosquito. Each stage is distinct, and is brought about by a sudden transformation. The new form comes from the shell of the previous form. The eggs are laid upon the water about two hundred to four hundred at a time. The larva and pupa live in water. They are, however, true air breathers, and have to come to the surface every minute or two to breathe. The length of a generation depends upon the temperature and other surroundings. Egg stage, eighteen hours to three days; larval, seven to sixteen days, and the pupal stage, two to five days. The average combined life of the three stages is twenty-four days. They pass the winter as adults. Hibernating mosquitoes may frequently be found during the winter months on the walls of barns, in cellars and garrets, beneath large leaves of trees, as the magnolia, and clustering in sheltered places beneath the eaves and in evergreen vines.

The mosquitoes are, normally, vegetarian in their diet, and do not at all require blood for their sustenance. Only a small proportion of them ever have an opportunity to satisfy this depraved taste, for countless numbers of them live and die in swamps which warm-blooded animals never penetrate; and, moreover, they are found in the uninhabited regions within the Arctic circle.

The *genera*, with which we most have to do, are the *Culex* and *Anopheles*. The males of both families are readily recognized by the antennæ, which are densely covered with long hairs, analogous to a bushy mustache; in the female, the hairs of the antennæ are short and very sparse. The mouth pieces of the male are not developed to the same degree of perfection for boring the human cuticle as are those of the female. They could not draw the blood if they would. They are, therefore, harmless, and the females are the members of the family who use their mouths most effectively, a not inhuman characteristic, though not a commendable one.

*Comparative Description of Culex and Anopheles.*—The *eggs* of the culex are found in an irregular raft-shaped mass, which is usually something like a pointed ellipse—somewhat convex below and concave above, all the eggs standing on end and closely applied side by side in from six to thirteen longitudinal rows, with from four to forty eggs in a row. The mass is about a fourth of an inch in length, and gray-brown in color. The eggs of the anopheles are scattered loosely upon the surface of the water, each egg lying upon its side. They are not attached, except that they naturally float close to each other. They are usually from forty to one hundred together.

*Larvæ* of the two forms differ in their structure, food, habits and customary position in the water. That of the culex has a much larger head than that of the anopheles. It spends much of its time feeding at the bottom; and when it is at the surface it holds its body at an angle of 45 degrees, thrusting up through the surface film its air tube, which projects from the body near the tail. The larvæ of the anopheles habitually remain at the surface. They hold their bodies parallel with and immediately below the surface film. The long fringes of the mouth parts of both species work violently and continuously, and cause a constant current toward the mouth, of particles floating on the surface of the water, which thus gradually converge to this miniature whirlpool and enter the alimentary canal. It has never been observed that male mosquitoes sing.

*Pupæ* of both forms breathe by means of tubes resembling ears on the top of their head. The pupa of the culex has not so large a head and hangs more vertically than the pupa of the anopheles.

The characteristic points of difference between the adult anopheles and culex are the presence of dark or brownish spots on the wings of the former, due to thicker growth of fine hairs in these regions. The palpi are as long as the proboscis. Thus observing the anopheles with the unaided eye or with a hand magnifying glass, we see five projections from its head, the central one the proboscis, and on either side the palpi of equal length with the proboscis. External to these are the hairy antennæ. In the culex, the palpi are only one-third as long as the proboscis. Another point of difference is that the song of the anopheles is several notes lower than that of the culex. Dr. Howard facetiously remarks that an interesting parallel to this is the fact that the villain in the play usually has a base voice.

It is said that the resting position of the anopheles, the body and the proboscis are in a straight line and almost vertical to the surface; but that the body of the culex rests parallel with the surface and the proposcis is bent downward, giving the insect more of a humpbacked appearance.

Mosquitoes breed wherever there is standing water, as swamps, rain-barrels, cisterns, holes in the ground, old tin cans, broken bottles, and, in fact, any place where water stands for two or three weeks, should be looked upon as a breeding-place. The larvæ of the anopheles have a special fondness for pools near a river bank and among the rocks where there are water-plants. I have not been able to find any larvæ ot the anopheles in rain-barrels or small pools in the city, but I did find some among the rocks on Belle Isle. Mosquitoes do not thrive in salt sea water, only in fresh or brackish water.

Knowing now the life histories and characteristics, we can intelligently direct our efforts at their extermination. Among the natural enemies of the mosquito are dragon flies. Not only as adults do they capture and devour adult mosquitoes, but their water-forms feed upon those of mosquitoes. Larva and pupa furnish juicy morsels to the other forms of animal life that dwell in the same pool. Little fish eat them. It was interesting to find, while recently looking among the rocks at Belle Isle, that in those holes where I found other forms of animal life, there were very few or no larvæ, whereas in other pools where there was not other animal life, they flourished abundantly. Leather-winged bats and other birds feed upon them. Perhaps the ladies would have less antipathy to the bats that set up such a commotion by their presence in a parlor on a summer's evening, if they knew that the bat's chief occupation in life was to make the insects "cease from troubling."

Many drugs have been used to keep away mosquitoes: Camphor rubbed upon the face or hands, or a few drops put on the pillow at bed-time; oil of pennyroyal and the oil of lavendar have this effect. Among the remedies for bites have been recommended glycerine, indigo and household ammonia. An old-fashioned way to kill mosquitoes is by means of a tin cup containing kerosene nailed to the end of a stick long enough to reach the ceiling. The cup is placed under a mosquito, which drops into the oil and dies.

The proper way to fight mosquitoes is by measures preventing their development. This is accomplished by abolishing the breeding places and by destroying the larvæ. In nearly every locality these measures are feasible and practicable, and there is no necessity for mosquitoes. Their presence may usually be attributable to ignorance or negligence on the part of the community or individuals. The three main preventive measures, as Dr. Howard says, are : the draining of breeding places, the introduction of small fish into fishless breeding places, and treatment of such pools with kerosene. Kerosene on the water prevents the larvæ and pupæ from projecting their air tubes through to the surface, and obstructing the tubes causes their death. These are three alternatives, any one of which may be used where there are reasons against the trial of others. The quantity of kerosene necessary is approximately, one ounce to fifteen square feet of water surface ; and ordinarily, the application need not be renewed for one month.

*Life History of the Malarial Parasite in the Mosquito.*—As you are all familiar with the development and the life cycle of the malarial plasmodium in the blood of animals, I will describe only the cycle of development of this parasite outside the human body, which so far has been found to occur only in the body of the mosquito of the anopheles variety. Instead of the parasite multiplying by the formation of spores as in the blood, the parasites, which are taken into the stomach of the anopheles, develop into two forms, one having projections or flagellæ, the other without them. The flagellæ break off from the one and fuse with the other form. The union of the two constitutes the fertilization of the organism. The fertilized organism now attaches itself to the wall of the stomach and penetrates the inner coat, locating itself just under the outer muscular coat of the stomach. It now increases rapidly in size, until eventually it becomes five times as large as at first. It is now known as a *zygote.* Upon the surface of the zygote, clear spaces, which are called centromeres, begin to appear. These centromeres are soon surrounded by minute, short, dark lines, which, under a very high power of the microscope, are seen to be spindle-shaped cells known as blasts. The blasts increase in size until they fill the entire zygote, obscuring the centromeres. Then the zygote bursts and the blasts are liberated through the muscular wall of the stomach into the body cavity of the mosquito. The blasts are very active, and penetrate into the tissue of the salivary duct, and so into the proboscis of the mosquito. With the saliva, or poison, they enter the blood of the next warm-blooded animal that the mosquito bites. The time required for the development of the blasts in the mosquito is about twelve days. It is supposed that the blasts enter the red blood corpuscles, and as the hyaline form of the malarial plasmodium, the development proceeds.

